# Learning 3D Bipedal Walking with Planned Footsteps and Fourier Series Periodic Gait Planning

**DOI:** 10.3390/s23041873

**Published:** 2023-02-07

**Authors:** Song Wang, Songhao Piao, Xiaokun Leng, Zhicheng He

**Affiliations:** School of Computer Science, Harbin Institute of Technology, Harbin 150009, China

**Keywords:** humanoid, footstep planning, reinforcement learning, gait phase

## Abstract

Reinforcement learning provides a general framework for achieving autonomy and diversity in traditional robot motion control. Robots must walk dynamically to adapt to different ground environments in complex environments. To achieve walking ability similar to that of humans, robots must be able to perceive, understand and interact with the surrounding environment. In 3D environments, walking like humans on rugged terrain is a challenging task because it requires complex world model generation, motion planning and control algorithms and their integration. So, the learning of high-dimensional complex motions is still a hot topic in research. This paper proposes a deep reinforcement learning-based footstep tracking method, which tracks the robot’s footstep position by adding periodic and symmetrical information of bipedal walking to the reward function. The robot can achieve robot obstacle avoidance and omnidirectional walking, turning, standing and climbing stairs in complex environments. Experimental results show that reinforcement learning can be combined with real-time robot footstep planning, avoiding the learning of path-planning information in the model training process, so as to avoid the model learning unnecessary knowledge and thereby accelerate the training process.

## 1. Introduction

There are various ground changes in the real environment, such as doorways, stairs, slopes, etc. Therefore, one of the key abilities of human-like robots is crossing inclined terrain and narrow corridors in a possibly adverse environment, and reducing the risk of failure to a minimum [1]. Bipedal robots need to complete tasks in complex working conditions while maintaining the robustness of the trunk balance at all times in high-dimensional motion planning, which requires robots to have reliable and stable motion controllers [2]. In the real environment, a single motion controller makes it difficult to meet this requirement because a single motion controller cannot meet the change of the landing point of the machine in a timely manner when walking. This will lead to the loss of balance in the robot and unexpected collisions with the objects in the environment. On the other hand, existing methods have a large number of footstep sequence planners. Additionally, these strategies include searching for global planning footsteps in 2D, which is usually more suitable for flat terrain and simpler avoidance.

Traditional footstep planners, after given the starting point and the target point, obtain the optimal path through the search algorithm. Then, they optimize the length of the centroid trajectory and the number of footsteps. Finally, the method of following the target trajectory produces the minimum number of footsteps needed to track the planned trajectory and reach the end pose [3,4].

In a dynamic environment, the robot needs to dynamically adjust its footsteps according to the changes in the local surrounding environment. The encoders on a wheeled robot can provide very accurate measurements of the distance traveled by the wheels, but they do not consider the robot’s orientation. On the other hand, a humanoid robot’s IMUs can provide information about the robot’s orientation and angular velocity. However, wheel encoders are more accurate when measuring linear distance traveled [4]. Therefore, it is necessary to have effective planning and replanning methods for a humanoid to quickly adjust the walking path to adapt to the changes in the dynamic environment.

Autonomous navigation in complex environments requires real-time motion generation and stability control in environments with obstacles, changing ground composition, and external disturbances. It will be widely used in practical applications only when leg robots can reliably walk and navigate complex scenes [5]. However, this will bring multiple challenges to bipedal robots in complex scenes: (1) the robot must solve the problem of autonomous motion generation in complex environments; (2) the robot must be able to respond quickly to dynamic changes in the environment or user input [6]; (3) the robot motion must be reliable and robust in the presence of unknown disturbances or modeling errors [7]; and (4) the robot must ensure the safety of the surrounding environment and itself. This requires the robot to have two qualities, namely the generalization of the motion model and the robustness of the motion. (1) The generalization of the motion model: when crossing or passing through unknown complex obstacles, or when walking with a larger step length, the motion model can effectively utilize the robot’s whole-body motion capability [8]. (2) Motion robustness: when the system needs to consider a large external disturbance as it is designed, the motion pattern needs to undergo a large change to achieve real-time stability recovery [9]. However, in existing research methods, the generalization and robustness of robot motion are often designed separately, without effective combination [10,11,12].

In recent years, with the development of reinforcement learning, non-model-based reinforcement learning algorithms have opened up a new field for legged robot motion control. Reinforcement learning policies can be trained to perform robot balancing, walking, and various complex operational tasks. One common solution is using a deep learning model to train and test the sample data from trials [13]. For robots, an important task is to be able to perform task operations according to human input instructions [14], such as moving according to the input direction. For navigation of complex environments, robots need to dynamically adjust their walking paths according to the perceived environment obstacles or semantic information. When walking S-shaped curves, flat or stair walking, and backward walking, humanoids should be able to maintain their own balanced standing state according to external input instructions [15]. Bipedal robots should also be able to easily switch between these different modes, ideally without switching to different controllers. Traditionally, the framework based on model-based control achieves this by combining footstep planning consisting of target foot positions and directions with a finite state machine (FSM). Robot footstep planning can significantly reduce the uncertainty of robot behavior, and the robot can understand when to control the landing point position, which improves overall safety [16,17].

In this paper, we propose a method for designing reinforcement motion policies for full-size robots so that humanoids can achieve omnidirectional and stair walking. The user input instructions are converted into the future two landing point positions and orientations, and the robot adjusts its joint angles according to its current state. A single motion policy and user input instructions can achieve the controlled walking task under different modes: forward and backward straight walking, curved walking, side walking, stair walking, in-place turning, and stationary standing.

The main contributions of this paper are as follows:

1. By introducing the periodic and symmetrical gait phase function of bipedal robot walking, this paper allows the robot to learn human-like motion without relying on dynamic capture information.
2. Omnidirectional locomotion on stairs and the ground is implemented, based on the footstep planner and orientation control. The landing point tracking locomotion is learned by reinforcement learning and leads to 3D walking based on the landing point planner.

## 2. Materials and Methods

### 2.1. Control Model Overview

**Figure 1:**
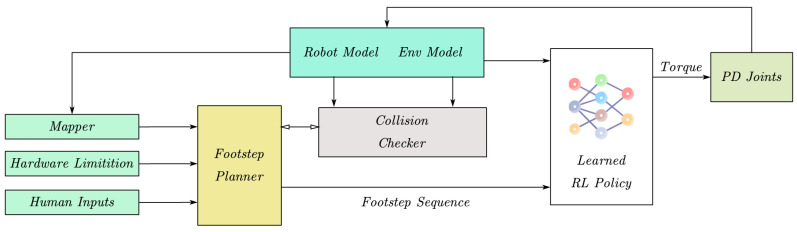
Hierarchical control structure.

The robot control layer is divided into the upper layer (HL) and the lower layer (LL) proportional derivative (PD) control. The upper layer controller frequency is 40 Hz for joint angle prediction, and the lower layer control frequency is 1000 Hz for converting joint position to joint torque. When the joint gain is low, the position tracking error of the lower layer PD controller will be very large. The policy network will include the output of the PD loop in the prediction range, and use the tracking error to generate internal interaction forces. This paper hopes that the controller can be combined with the planner, and the planner outputs the target landing point and orientation angle to HL controller. The planner dynamically generates landing points based on the change of the environment, and then follows the robot, as shown in Figure 1.

### 2.2. Planning System

#### 2.2.1. Map Grid

In 3D environment, real-time path planning based on a heuristic guided search requires a large amount of computing resources. To reduce the computational cost of the path search, the complex environment information is reduced to a low-dimensional grid map, and then the path planning search is performed [18]. This greatly reduces the computation while retaining a certain 3D environment surface feature. The ground is divided into grids of the same size, and each grid is marked as to whether it can be freely walked, stepped on or crossed. When the grid is completely occupied, for example, with a wall, the robot will choose the detour strategy.

To effectively build terrain maps and perform collision detection, the following assumptions are made:

1. The working ground environment of the robot is divided into a floor that can be stepped on and obstacles;
2. The floor is horizontal and will not be tilted, and other obstacles are treated as obstacles;
3. There will be no more than one floor at the same location; and
4. The robot can distinguish between the floor and obstacles through its own sensors.

Based on the above assumptions, two maps are constructed; grid terrain maps M. M contains info about the horizontal plane, obstacle type and height, which is usually obtained by the robot’s depth camera or 3D laser radar scan and is divided into a semantic map by probability. M is represented as

(1) M(x,y)=(c,h,d)

where x∈X,y∈Y, c∈C={floor,obstacle,stair,border,unkown},h∈R represents the height of the floor or obstacle, d∈R represents the distance interval, that is, the Euclidean distance of the nearest obstacle to the current grid.

According to the ground grid map M, the navigation map is constructed to save the type of grid map and the distance information of the nearest neighbor obstacle. Let N(x,y) represent the neighborhood area of (x,y). $h^{\prime }$ is the height of any grid $\left(x^{\prime },y^{\prime }\right)$ in the vicinity with a height difference of $\left|h-h^{\prime }\right|$. $\Delta \hat{h}$ is the maximum height difference of the obstacle from the ground

(2) $$\Delta \hat{h}=\underset{\left(x^{\prime },y^{\prime }\right)\in N\left(x,y\right),M\left(x^{\prime },y^{\prime }\right)=\left(\mathrm{floor},h^{\prime }\right)}{\max}\left|h-h^{\prime }\right|$$

According to the height difference $\Delta \hat{h}$, the type of obstacle is judged, that is,

(3) $$\begin{array}{rr} t\left(\mathrm{floor}\right) & =\{\begin{array}{ll} \;\mathrm{floor},\; & \Delta \hat{h}\leq d_{\mathrm{floor}\;}\\ \;\mathrm{stairs},\; & d_{\mathrm{floor}\;}<\Delta \hat{h}\leq d_{\mathrm{stairs}\;}\\ \;\mathrm{border},\; & d_{\mathrm{stairs}\;}<\Delta \hat{h} \end{array} \end{array}$$

where $d_{floor}$ is the maximum height of normal walking, and $d_{\mathrm{stairs}\;}$ is the maximum height of walking stairs. Since the robot needs to keep a certain safe distance between the stairs when walking, the boundary between the stairs and the horizontal ground needs to be handled separately. When doing path planning in a stair environment, the optimal path is selected by traversing the cost value.

**Figure 2:**
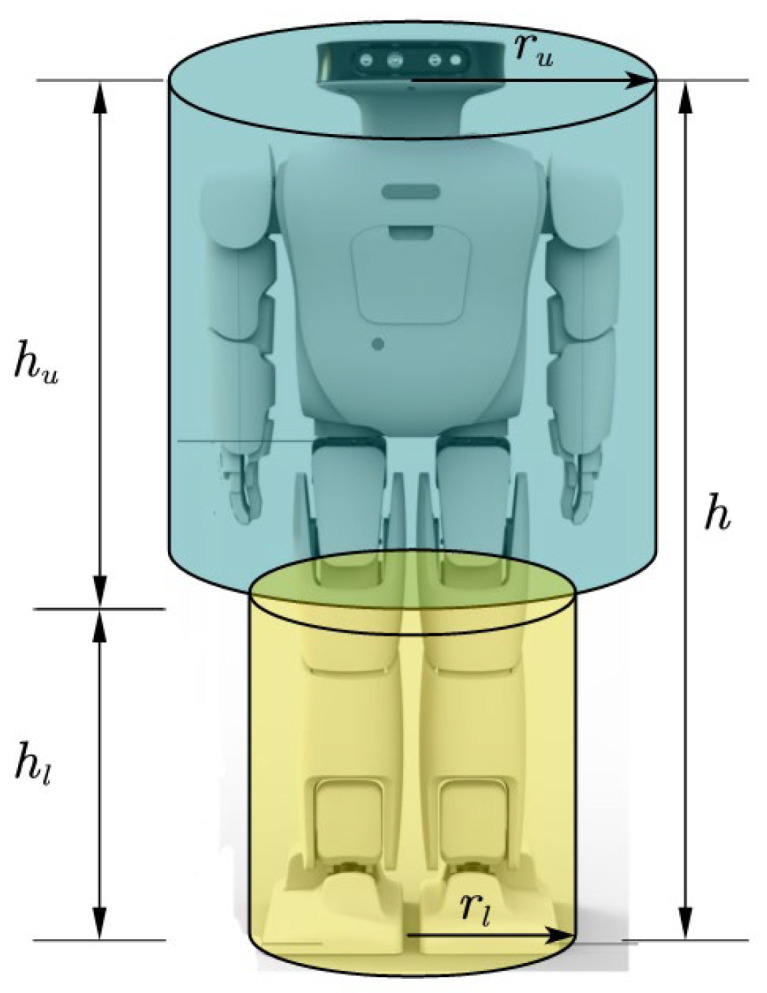
Robot shape multi-cylinder model.

The height difference between grids is defined as $\Delta h^{\prime }\left(x^{\prime },y^{\prime }\right)=h^{\prime }-h$, as shown in Figure 2; due to the difference in the collision volume radius of the upper and lower halves of the robot, the distance between the robot boundary and the grid is judged according to the height difference between the target grid and the current grid of the robot, that is,

(4) $$d^{\prime }\left(x^{\prime },y^{\prime }\right)=\{\begin{array}{ll} e-r_{l}, & 0\leq \Delta h^{\prime }\left(x^{\prime },y^{\prime }\right)<h_{l}\\ e-r_{u}, & h_{l}\leq \Delta h^{\prime }\left(x^{\prime },y^{\prime }\right)\leq h\\ \infty , & \;\mathrm{otherwise}\; \end{array}$$

where $e=\sqrt{{\left(x^{\prime }-x\right)}^{2}+{\left(y^{\prime }-y\right)}^{2}}$ is the Euclidean distance between grid $\left(x^{\prime },y^{\prime }\right),$ and (x,y), $r_{l}$, $r_{u}$ is the radius of the collision cylinder of the lower half and upper half of the robot. $h_{l}$ is the height of the upper half of the robot, and $h_{u}$ is the height of the lower half of the robot. According to the above definition, when the robot walks on different height grids, it needs to ensure that the whole body of the robot will not collide with the obstacle.

The distance of the grid (x,y) to the nearest obstacle or boundary is defined as d(x,y), that is

(5) $$d\left(\mathrm{floor}\right)=\underset{\left(x^{\prime },y^{\prime }\right)\in X\times Y,N\left(x^{\prime },y^{\prime }\right)=\left(c^{\prime },\cdot \right),c^{\prime }\in \left\{\mathrm{obstacle},\;\mathrm{border}\right\}}{\min d^{\prime }\left(x^{\prime },y^{\prime }\right)}$$

In actual calculation, the nearest neighbor point can be searched outward from the grid point as the center until the boundary or obstacle is found, and the search ends.

Let w represent the grid accuracy, and the grid be represented by $c_{x,y}$, which can represent the floor, stairs, door threshold, narrow beam and obstacle, etc., which correspond to the normal walking behavior of the robot, stepping, crossing and detouring, etc. On the defined grid map M, the 3D trajectory from the starting point to the end point is represented by

(6) $$P^{\left(i\right)}=\left\{\overset{n}{\underset{j=1}{\sum }}p_{j}|H_{c}\leq H_{limit},W_{x}>l_{\mathrm{foot}},W_{y}>b_{\mathrm{foot}\;}\right\}$$

where $H_{limit}$ is the grid’s maximum height limit. $W_{x}$, and $W_{y}$, are length of the reachable area of the landing foot in the *x* and *y* directions, respectively. $l_{foot}$ and $b_{foot}$ are the width and length of the robot’s foot, respectively. $p_{j}$ is the foot trajectory. The different motion trajectories $P^{\left(i\right)}$ represent the different motion capabilities of the biped robot.

#### 2.2.2. Path Planning

**Figure 3:**
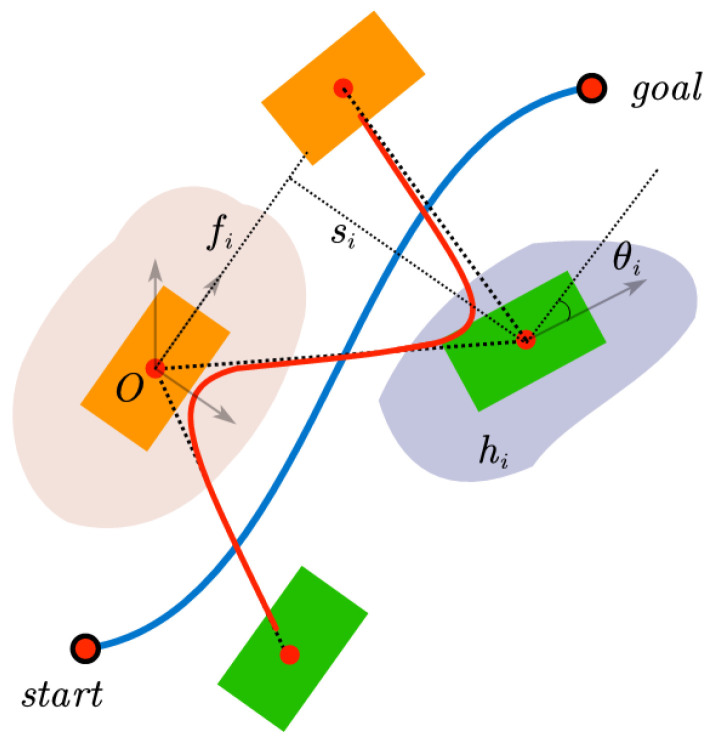
Footstep definition.

Compared with [19], which is used to identify the position of the next step by dynamically adjusting the foot in the next two steps, it is impossible to obtain global planning information, and the path planning information needs to be learned completely. By introducing the path planning module in the robot’s learning kinematics, the learning speed of the robot can be accelerated, and the motion capability of the robot can also be improved. In 3D environment, the state of the ith landing point is defined as

(7) $$s_{i}={\left[\begin{array}{llllll} t_{i}^{SS} & t_{i}^{ds} & f_{i} & s_{i} & h_{i} & \theta_{i} \end{array}\right]}^{T}$$

where $t_{i}^{SS}$ and $t_{i}^{DS}$ represent the time length of single support phase and double-support phase, respectively. The feasibility of the footstep generated by the gait pattern generator is affected by the walking cycle of the landing point. Therefore, the walking cycle information is included in the definition of the footstep. $f_{i}$, $s_{i}$, $h_{i}$, $\theta_{i}$ represent the forward walking length, lateral walking length, foot height and walking direction angle in the local coordinate system of the supporting foot, as shown in Figure 3.

The robot walks through transitions between states. Transitions between states require actions $a\in A:s^{\prime }=t\left(s,a\right)$, where the next state is $s^{\prime }$, and the state transition trajectory is searched for in the optimal state transition. We may define the state transition cost function $c\left(s,s^{\prime }\right)$ as

(8) $$c\left(s,s^{\prime }\right)=\left(x,y\right),\left(x^{\prime },y^{\prime }\right)+k$$

where k is the cost constant required to execute a step, ensuring that the minimum number of steps is used to reach the target point. The state $s\mapsto s^{\prime }$ transition forms a sparse connected grid graph, where the next landing point corresponds to the motion primitive in path search. The biped robot achieves the trajectory movement of the center of mass by changing the touchdown points of the left and right feet. By referring to the Equations (4) and (5), the footstep planning intends to walk without collision with the surrounding environment and within the range of the foot height that can be reached. This paper uses the $A^{*}$ algorithm based on search to optimize and solve the footstep planning in [20].

### 2.3. Walking Pattern Generation Based on Reinforcement Learning

In the method framework of this paper, the position of the touchdown point consists of a 3D coordinate point and an orientation angle θ, which corresponds to the target position of the foot and the yaw of the robot root. By attaching the heading vector to the touchdown point, the complex walking path of the robot for example, in lateral walking and in-place turning, can be simplified. This ordered sequence generation is called footstep planning. For the forward straight walking of the robot, only the touchdown point positions on both sides of the trajectory need to be generated in turn, and the root direction angle faces forward and remains unchanged. The step length and the distance between the two feet when standing need to be adjusted according to the robot model. An excessively large step length and distance may cause the robot to be unstable. Similarly, for backward walking, the footstep planner needs to generate the touchdown point positions on both sides of the trajectory, and the root direction angle faces forward and remains unchanged. For stationary standing, the touchdown point has only one group, and the root position remains at the origin (that is, $T_{1}=T_{2}=0$). For the robot’s side-to-side movement, the root yaw angle remains unchanged, and the touchdown point moves linearly along with the horizontal direction.

For the stair stepping task, the touchdown point needs to change the height in the z direction, and the up and down stairs can be trained through curriculum learning. First, the robot is trained on the plane at z=0 to learn the ability to stand and walk steadily on the ground. Then the stair step is fixed to be equal to the step length, which ensures that the observed target step is exactly in the middle of the stair step, and the z exploration and learning of complex stair walking skills are gradually increased.

For the robot’s curved walking, the footstep planner of this paper uses the 2D bipedal walking footstep group and planner based on search [20] for the trajectory of the humanoid robot. The planner’s input is the grid map of the uneven environment, and the initial and target positions (x,y,θ). From the starting position, 1000 footstep plans are randomly sampled from (0,−1,−π/2) to (0,1,π/2) in the blank map. Obstacles are generated on the grid map to generate sharper and more complex trajectories. The robot’s reinforcement learning policy outputs actions by observing the footstep position, the root orientation angle, and the robot’s current action frame. When the robot successfully reaches the target position, the observation window slides. The motion step of the kth step is replaced by k+1, and a hit delay and hit radius are set to ensure that the swinging foot has enough time and radius range to reach the target footstep.

### 2.4. Gait Period Segmentation Based on Fourier Series

For the upper-level control algorithm that requires the gait pattern to be a real-time variable rather than a fixed walking length and step height, according to [21], the bipedal walking of the robot can be divided into two phases according to the change of zero moment point (ZMP) and center of gravity (CoG) of the left and right feet i.e., double-support phase (DSP) and single-support phase (SSP). The traditional bipedal walking gait generator LIMP can only fix the gait walking period, and cannot adjust the gait period parameters in real time according to the control input requirements. Fourier series can effectively approximate various periodic continuous functions. Compared with spline estimation methods, Fourier series can generate smoother curves [22]. The Fourier series method can approximate the ZMP trajectory of the robot walking in the frequency domain.

**Figure 4:**
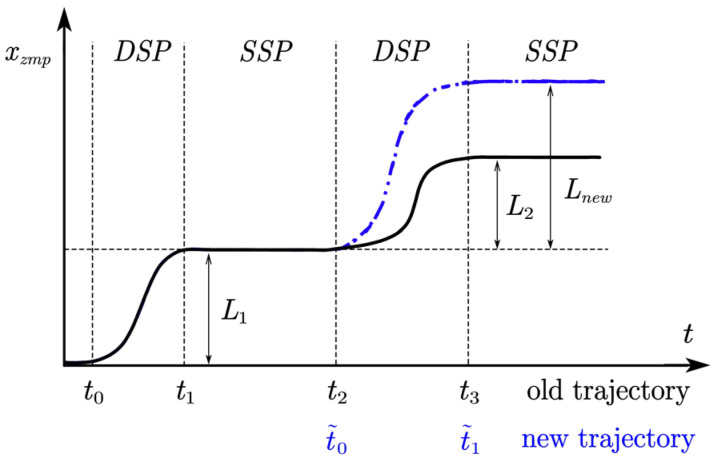
Phase segmentation based on ZMP trajectory.

The walking phase of the robot can be divided into three phases based on the ZMP trajectory. In the *x*-axis direction, the ZMP trajectory of the robot walking two steps is shown in Figure 4. The robot starts from $t=t_{0}$ and enters the first double-support phase, and ends at $t=t_{1}$. Then, the gait is in the single-support phase between $t_{1}$ and $t_{2}$. The gait enters the double-support phase again between $t_{2}$ and $t_{3}$.

We can assume that the ZMP trajectory $P_{j}\left(t\right)$ of each phase is a periodic function, and the period length is $2L_{j}$, $L_{j}=t_{j+1}-t_{j}$, and the Fourier series representation of the ZMP periodic function of each phase is represented by Equation (9).

(9) $$\begin{array}{c} P_{j}\left(t\right)=a_{0}^{j}+\overset{N}{\underset{i=1}{\sum }}a_{i}^{j}\cos\left(iw_{j}t\right)+b_{i}^{j}\sin\left(iw_{j}t\right)\\ w_{j}=\frac{\pi }{L_{j}} \end{array}$$

where N is the order of the Fourier series, j=1,2,3,… represents each segment, and the calculation method of the coefficients $a_{0},a_{i},b_{i}$ is as follows:

(10) $$\begin{array}{c} a_{0}^{j}=\frac{1}{2L_{j}}\int_{-L_{j}}^{L}P{\left(t\right)}_{j}dt\\ a_{i}^{j}=\frac{1}{L_{j}}\int_{-L_{j}}^{L_{j}}P\left(t\right)\cos\left(\frac{i\pi }{L_{j}}t\right)dt,i=1,2,\ldots ,N\\ b_{i}^{j}=\frac{1}{L_{j}}\int_{-L_{j}}^{L_{j}}P\left(t\right)\sin\left(\frac{i\pi }{L_{j}}t\right)dt,i=1,2,\ldots ,N \end{array}$$

The analytical solution of the CoM trajectory x(t) can be obtained by a linear inverted pendulum model (LIPM). By adjusting the period time $t_{0},t_{1},t_{2},t_{3}$, it is possible to generate a variable double-support phase and a single-support phase time interval motion. The Fourier series guarantees the periodicity and continuity of the ZMP trajectory, avoiding the instability of the velocity caused by the abrupt change of the position. Therefore, the segmented method can guarantee the continuity of the ZMP trajectory, and also guarantee the periodicity of the ZMP trajectory, so as not to consider the continuity problem of the starting and ending of the gait cycle. Since the robot walking is a periodic motion, the clock information is defined as the coefficient of the periodic reward. The clock signal can be expressed by a single scalar phase $\phi ={\dot{P}}_{j}\left(t\right)$ and the time signal can be expressed by the phase of the periodic motion by double mapping ϕ so that it changes between 0 and 1. The robot state vector consists of 8D of external state vector and 2D of clock signal. The external vector consists of 3D coordinates of the next 2 foots landing points and 1D target orientation angle θ $T_{1}=\left[x_{1},y_{1},z_{1},\theta_{1}\right]$, $T_{2}=\left[x_{2},y_{2},z_{2},\theta_{1}\right]$. The orientation angle θ is the target orientation angle of the root. Although using two foots landing points may result in a performance decrease, more prediction steps will not provide more information [19].

### 2.5. Observation Space and Action Space

**Observation Space:** The robot simulation model contains 21 joints controlled by torque, and the torque output range of different types of joints is different. In order to avoid the output range of torque of different types of joints being different, which leads to the unstable output of the policy, the torque output range of all joints is normalized to [−1,1]. The robot state observation space contains the joint angles and velocities, the centroid trunk posture, the centroid linear velocity and angular velocity. At the same time, the state space also contains the height of the trunk from the farthest foot bottom, and the contact situation between the foot bottom and the ground. Therefore, it is naturally important for the external foot trajectory planner responsible for calculating the relative foot trajectory to estimate the pose of the robot in the world coordinate system.

**Action Space:** The output of the policy consists of the target joint positions of the leg that is under-driven (each robot contains 12 leg degrees of freedom). The predicted joint positions of the policy network will be added to the posture joint angles of the robot when it presents a squatting state, and then sent to the PD controller at the bottom layer.

### 2.6. Reward Function Design

**Figure 5:**
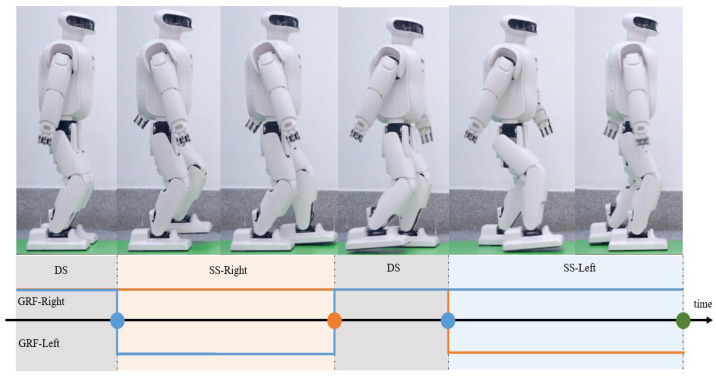
Symmetric gait cycle.

**Bipedal Walking:** Bipedal animals have the characteristics of symmetry and periodicity in walking, and their walking switches between double-support phase (DS) and single-support phase (SS). In the double-support phase, the robot’s two feet are in contact with the ground at the same time; in the single-support phase, the robot’s single foot is in contact with the ground, and the swing foot swings at a certain speed under control. In order to encode the walking behavior pattern of the robot’s two feet, this paper uses the periodic reward function structure proposed in [23]. The gait cycle is divided into two SS and two DS phases of fixed length, as shown in Figure 5. In the single-support phase, the supporting foot is in static contact with the ground without relative sliding, and the swing foot swings at a certain speed under control. The change trajectory of the robot ZMP point is known (9), and the structural information of the robot is known, that is, the length of each link and the position of the joint freedom degree. According to the robot’s forward kinematics, the trajectory of the left and right foot bottoms can be obtained. And the velocity cycle function of the landing point of the two-foot robot can be obtained by taking the derivative of the trajectory of the foot bottom, as shown in Figure 5.

Phase indicator functions are used to describe the periodicity of the robot’s bipedal walking. $I_{left}^{grf}\left(\phi \right),I_{right}^{grf}\left(\phi \right),$ respectively represent the force acting on the left and right foot bottoms with the ground, $I_{left}^{spd}\left(\phi \right),I_{\mathrm{right}\;}^{spd}\left(\phi \right),$ respectively represent the speed of the left and right feet, as shown in Figure 5. In the single-support phase, the function $I_{*}\left(\phi \right)\in \left[-1,1\right]$ stimulates the swing foot to swing at a higher speed, and punishes the ground reaction force exerted on the swing foot. At the same time, the relative sliding between the supporting foot and the ground is punished. Additionally, the ground reaction force exerted on the supporting foot is stimulated. In the double-support phase, the function $I_{*}\left(\phi \right)\in \left[-1,1\right]$ stimulates the ground reaction force exerted on the supporting foot, and punishes the speed of the supporting foot.

The function that controls the ground reaction force and swing speed of the two feet is represented as

(11) $$\begin{array}{rr} & r_{grf}=I_{left}^{grf}\left(\phi \right)\cdot F_{left}+I_{right}^{grf}\left(\phi \right)\cdot F_{\mathrm{right}\;}\\ & r_{spd}=I_{left}^{spd}\left(\phi \right)\cdot S_{left}+I_{right}^{spd}\left(\phi \right)\cdot S_{\mathrm{right}\;} \end{array}$$

As shown in Equation (11) and Figure 5, the normalized ground reaction force ($F_{left}$, $F_{right}$) and speed ($S_{left}$, $S_{right}$) of the two feet are constrained by the function $I_{*}\left(\phi \right)$ to determine the size of the reward. For example, when ϕ is in the single-support phase of the gait cycle, and when the value of $I_{right}^{grf}\left(\phi \right)$ is close to 1, the value of $I_{left}^{grf}\left(\phi \right)$ is close to -1, that is, a larger $F_{left}$ will receive a smaller reward, and a larger $F_{right}$ will receive a larger reward. The left foot swings and the right foot plays a supporting role.

For the static standing action, the double-support phase will be extended to the entire gait cycle, so the landing point of the static standing will not move, that is, $T_{1}=T_{2}=0$. When the robot moves, in addition to the periodic reward function of the robot, it is also necessary to ensure that the next landing point of the robot and the planned point are as close as possible. Therefore, the step reward function is defined as

(12) $$r_{\mathrm{step}\;}=k_{\mathrm{step}\;}\exp\left(-d/k_{d}\right)$$

where d is the straight line distance between the target point and the center of the landing foot. By changing the value of $k_{step}$ and $k_{d}$, the sensitivity of the step reward can be adjusted. This value is affected by the size of the robot’s foot and the length of the two feet. For different robot models, the hyperparameters need to be adjusted. Only when the front foot of the robot touches the ground will the step reward be calculated. When the robot is in the air phase, the reward value will not be calculated if one foot is on the ground. Since the initial stage of training has a large error between the landing point and the target point, the exponential function is introduced to make the reward value increase with the decrease of the error. On the one hand, the change of the gradient of the reward function is reduced. On the other hand, it encourages the robot to approach the target point as soon as possible.

The reinforcement learning algorithm finds it difficult to optimize the sparse reward such as $r_{step}$. Subsequently, the process reward function is introduced, that is, stepreward; in addition to the robot periodic reward function, it is necessary to consider whether the robot can walk according to the target point and the target orientation. The step reward function needs to be combined with two aspects: landing point overlap reward and progress reward. The landing point overlap reward encourages the robot to land the landing point on the target point to be reached. When the foot reaches a certain radius range with $T_{1}$, the reward value is generated. The progress reward value encourages the robot to move the trunk towards the next target point (2D plane).

Let the distance between $T_{1}$ and the nearest foot be $d_{foot}$, and the distance between the root and the connecting rod be $d_{root}$, and the step reward value is represented as

(13) $$\begin{array}{rr} r_{step}=k_{hit}\cdot \exp\left(-d_{\mathrm{foot}}/0.25\right)+ & \left(1-k_{hit}\right)\cdot \exp\left(-d_{\mathrm{root}}/2\right) \end{array}$$

In addition, the orientation reward function encourages the robot root attitude quaternion q to approach the target quaternion $\hat{q}$, that is,

(14) $$r_{\mathrm{orient}}=\exp\left(-10\cdot \left(1-{\left(q⊕\hat{q}\right)}^{2}\right)\right)$$

where the operator ⊕ represents the inner product of the quaternion.

**Other reward functions for bipedal walking:** In the condition of missing action capture reference action, the robot learning to imitate human walking also needs to consider comprehensively the limited joint output torque condition, so that the body maintains a specific posture characteristic and undergoes optimal energy loss. Therefore, this paper refers to [19,24] to design the reward function, and the calculation method is as follows:

(15) $$r_{\mathrm{addition}}=r_{\mathrm{height}\;}+r_{\mathrm{upper}\;}+r_{\mathrm{energy}\;}+r_{\mathrm{alive}\;}$$

In addition to the above reward functions, the robot trunk height $h_{root}$ should be close to the target height ${\hat{h}}_{\mathrm{root}\;}$, as shown in the following

(16) $$r_{\mathrm{height}}=\exp\left(-40\cdot {\left(h_{\mathrm{root}}-{\hat{h}}_{\mathrm{root}\;}\right)}^{2}\right)$$

To make the robot’s upper body maintain an upright state, let the robot’s head projection on the horizontal plane be ${}^{x},yp_{head}$, and the root projection on the horizontal plane be ${}^{x},yp_{root}$. To constrain the robot’s upper body swing, the upper body posture reward function is constructed:

(17) rupper=exp(−10·‖pheadx,y−prootx,y‖2)

In order to reduce the energy consumption of bipedal walking and improve the efficiency of walking, the output torque and action at the current time and the output torque and action at the previous time are used to make $r_{\mathrm{energy}\;}$ smaller, which can better guarantee the efficiency of the robot.

(18) $$r_{\mathrm{energy}}=-\frac{1}{N_{j}}\underset{j}{\sum }\left|a_{j}\cdot v_{j}\right|-\frac{1}{N_{j}}\underset{j}{\sum }{\left|a_{j}\right|}^{2}$$

where $N_{j}$ is the number of joints, $a_{j}$ is the normalized torque of joint j, and $v_{j}$ is the joint velocity.

Finally, the robot’s survival reward $r_{alive}=1$ is defined, and the robot’s survival reward is 1 when the current round is not over, which can make the robot no longer eager to obtain a high reward value and maintain motion balance.

### 2.7. Curriculum Learning Strategy

Except for the reward function, the initial state of the robot and the end condition of the reinforcement learning training round also have a significant impact on the final behavior, which can effectively prevent the robot from sampling on the wrong sampling distribution. It is similar to the class imbalance of supervised learning.

Firstly, the robot’s initial state is kept in a stable standing state, and can be maintained in this state without external force interference, which is an initial condition. For humanoid robots, it is usually an initial state of squatting, and then by adding noise to this state, the policy can adapt to different robot initial states, which is critical for deploying the model on physical robots. Since the policy does not input the robot’s yaw angle, the robot’s initial orientation does not need to add noise.

The conditions for early termination of the round need to be carefully designed, because the balance state of the robot is very difficult to recover. When the root height of the robot is lower than the threshold value, or when a part other than the foot touches the ground, the round is terminated in advance. At the same time, when the round reaches the maximum number of steps, the round ends automatically.

In previous work, curriculum learning was used to solve the problem of getting stuck in local optima when learning difficult tasks. For the robot walking task, the robot was first trained to keep still standing in place, then learned flat walking, and finally stair walking.

To let the robot learn to walk up and down stairs, and learn other complex tasks, a sampling distribution function is defined. First, the policy is only trained on the flat ground, and then the target of the flat curve walking is manually generated. When the number of training iterations accumulates to a certain extent, the position of the landing point support point in the physical engine is changed (the height of the landing point is 0 ±10 cm), and now the index value of the curriculum learning still needs to be manually adjusted by experience.

## 3. Results

**Figure 6:**
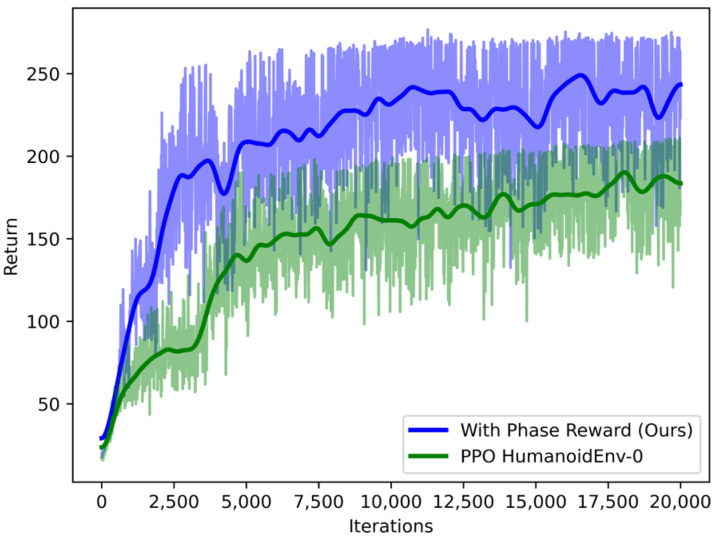
Evaluation curves on environment with the Humanoid robot. For clarity, we plot mean reward performance averaged over 20,000 samples.

In this paper, the model is trained and experimentally verified on the humanoid and Roban robot models. In order to verify the effectiveness of the proposed robot walking policy learning method, the actor and critic networks are first trained based on a proximal policy optimization (PPO) algorithm, and the network structure uses the MLP structure. The deep reinforcement learning training uses the central processing unit (CPU) of the computer AMD EPYC 7543 (128) @ 2.800 GHz, the graphics processor are 2 RTX3090 from Nvidia, and the memory is 128 GB. As shown in Figure 6, it is known that the convergence of the method based on the gait phase function reward policy in Section 2.6 is faster than that of the pure walking training method without $r_{grf}$ and $r_{spd}$. When the training time reaches 5000 times, the robot can perform relatively complete walking actions on non-stable ground.

**Figure 7:**
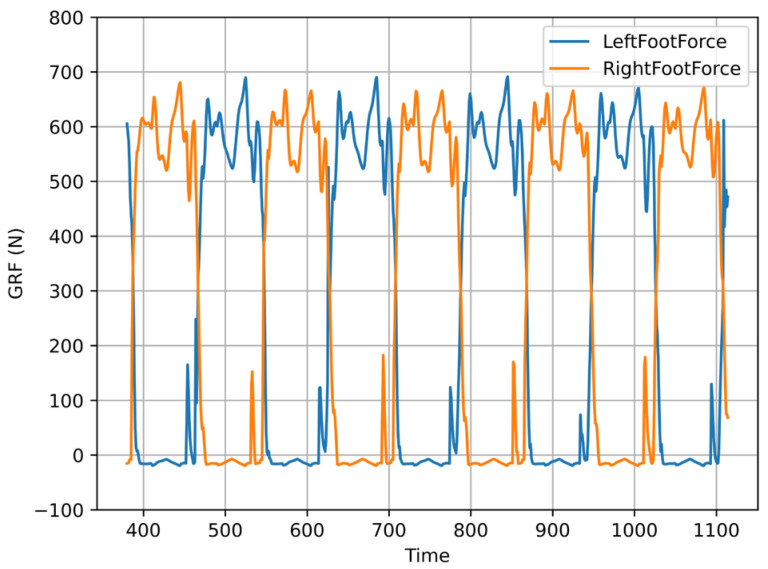
Left and right foot ground pressure (GRF) change curve.

Through the measurement of the vertical ground reaction force (GRF) value of the foot pressure sensor during walking, the gait can maintain symmetry and periodicity when walking on the flat ground. As shown in Figure 7, the policy has learned the approximate periodicity and symmetry switching gait without any prior information, which is consistent with the gait characteristics of the bipedal motion pattern in nature. At the same time, it can be found that in most cases, the robot keeps the single-leg landing state, that is, the single-support phase. In this state, the robot’s center of mass position motion is stable, but there is still an imbalance problem of the single foot GRF force size. This problem will be further solved in subsequent work. It can be found that the gait model obtained by learning can contain the modal mode of LIPM, and this way, the gait is more flexible, with better generalization.

**Figure 8:**
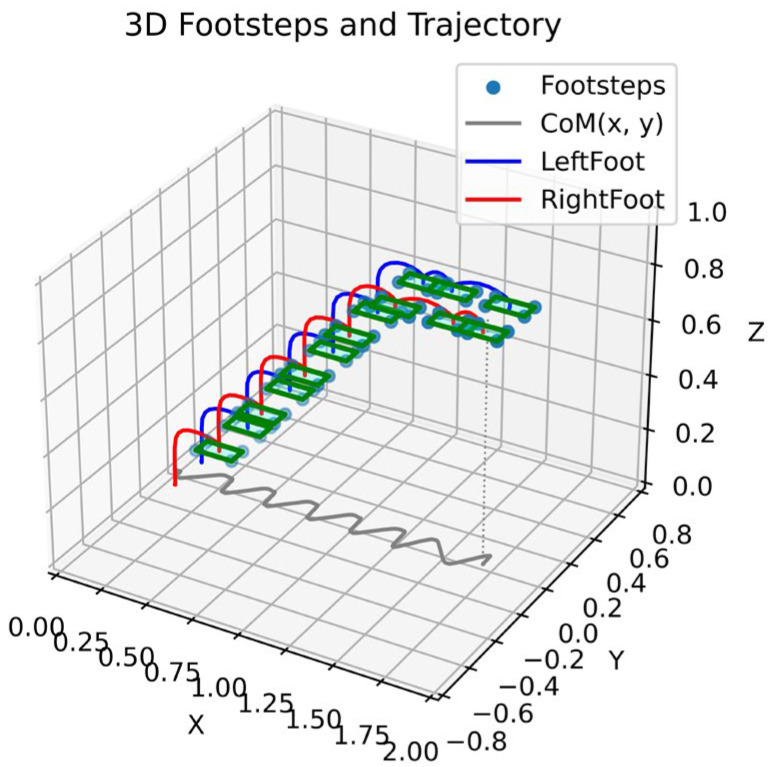
Robot steps on the landing point, foot and center of mass projection trajectory.

With the learning of the flat ground walking policy, the height of the stairs gradually changed as the training increased, so that the robot can walk on the stairs. Figure 8 shows the footprints, center of mass and left and right foot space trajectories of the robot when walking on the non-stable ground stairs. From the experimental results, it can be seen that all motion trajectories can finally converge to the target point, which shows that the learned motion model has the characteristic of global asymptotic stability.

**Figure 9:**
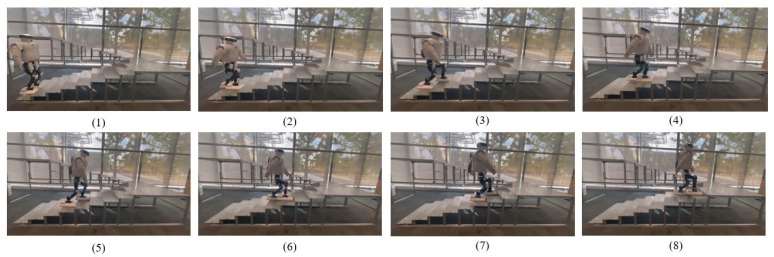
Roban robot climbing up staircase with learned walking model.

However, the simulation environment cannot simulate the physical parameters in the real environment, such as sensor measurement value error, the friction coefficient between each contact surface, etc.; the reinforcement learning policy still has a great challenge in applying these to the real physical robot. This paper uses domain randomization [25] to add Gaussian random noise to the observation space and action space in Section 2.5 during the training process to improve the generalization of the motion model. To verify the feasibility of the motion gait learned by the method in the physical sample machine platform, this paper extracts the policy model generated by the policy network in the simulation, and conducts the gait experiment on the sample machine. The learned model is applied to the Roban robot for experimental verification. As shown in Figure 9, the robot can maintain symmetry and periodicity when walking on the stairs, and the projection trajectory of the robot’s center of mass and foot is similar to the trajectory in the simulation environment when walking on the stairs, which shows that the learned model has good generalization.

From the experimental results, it can be seen that one of the advantages of neural network controllers in engineering applications is that they can select suitable models from multiple models generated during training. Although most of the models have poor adaptability to the environment, they can still be deployed on the experimental robot through randomization and other methods to find models that meet the conditions.

## 4. Conclusions

This paper introduces a robot gait learning method based on reinforcement learning. By introducing the landing point planning and the phase function of the bipedal walking, the modal pattern containing LIPM is learned, and the gait is more flexible and has better generalization. By introducing the trajectory planning method, the landing point trajectory is tracked to accelerate the learning efficiency and the utilization rate of the sampling data, and the convergence is completed faster. The learned model can realize complex motions such as standing, walking, curved walking and climbing stairs for bipedal robots.

Unlike traditional whiteboard learning, the method proposed in this paper can apply the model to the physical bipedal robot through domain randomization. By mounting the foot pressure sensor, joint angle sensor and IMU information on the robot, the vertical position of the landing point planner is changed in order to accomplish the robot walking on the stairs. The introduction of the phase function further indicates the important characteristics of the gait walking for the model training, and the learning direction is better constrained by the reward function to better achieve the training of the walking task. This study provides a more flexible learning method to help robots complete tasks in complex environments.

## Data Availability

The data used to support the findings of this study are available from the corresponding author upon request.
